# Fascial Plane Blocks With Glucocorticoids or Liposomal Bupivacaine Versus Local Infiltration for Laparoscopic Non-donor Nephrectomy: A Propensity Score-Weighted Study

**DOI:** 10.7759/cureus.66005

**Published:** 2024-08-02

**Authors:** Kaivon Sobhani, Mark Hocevar, Stephanie Hanchuk, Benjamin Press, Zili He, Hung-Mo Lin, Jinlei Li

**Affiliations:** 1 Anesthesiology, Yale School of Medicine, New Haven, USA; 2 Urology, Yale School of Medicine, New Haven, USA; 3 Yale Center for Analytical Sciences (YCAS), Yale School of Medicine, New Haven, USA

**Keywords:** nephrectomy, fascial plane block, methylprednisolone acetate, dexamethasone, liposomal bupivacaine

## Abstract

Study objective

The purpose of this study is to investigate the analgesic efficacy of ultrasound-guided fascial plane blocks (FPBs) versus local infiltration in patients undergoing laparoscopic non-donor nephrectomy. This study specifically compares the efficacy of FPBs with liposomal bupivacaine (LB) versus FPBs with dexamethasone sodium phosphate (DXP) and methylprednisolone acetate (MPA) versus surgical site local anesthetic infiltration without FPBs.

Design

This is a retrospective cohort study conducted over a five-year period (January 2018-December 2022).

Setting

The study was conducted in a tertiary care, academic, multi-hospital healthcare system.

Participants

Patients who underwent elective radical or partial laparoscopic non-donor nephrectomy were included in the study.

Intervention

Patients either received preoperative FPBs without intraoperative surgical site local anesthetic infiltration or received surgical site local anesthetic infiltration without FPBs (n = 141) at participating hospitals.

Measurements

The primary endpoint of this study was postoperative opioid use, measured as oral milligram morphine equivalents (MME). Secondary endpoints included postoperative pain scores, length of hospital stays, and significant adverse events within 30 days. The impact of medications utilized in FPBs was determined by univariate and multivariable analyses with covariates balancing propensity score weighting.

Main results

Patients undergoing non-donor laparoscopic radical or partial nephrectomy who received FPBs with bupivacaine or ropivacaine plus glucocorticoids DXP and MPA were more likely to be opioid-free 24-48 hours postoperatively compared to those who received FPBs with LB or surgical site local anesthetic infiltration without FPBs (40.5% vs. 30% vs. 13.9%, respectively; p = 0.017). Patients who received FPBs with glucocorticoids also reported the lowest pain scores at rest and with activity 0-12 hours postoperatively as compared to patients who received LB or local infiltration (p = 0.006 and p = 0.014, respectively). Additionally, patients who received FPBs with glucocorticoids received over 30% fewer opioids during the first 48 hours postoperatively compared to patients who received surgical site local anesthetic infiltration alone (30 MME vs. 44 MME, respectively). However, there was no significant difference in total opioid use during the first 48 hours postoperatively between patients who received FPBs with bupivacaine plus glucocorticoids and those who received FPBs with bupivacaine plus LB (mean ratio: 0.91, (95% CI: 0.05 ~ 15.97); p = 0.948). There was also no difference in the length of hospital stays or rate of adverse events between the groups.

Conclusion

Perioperative FPBs for non-donor laparoscopic nephrectomy using glucocorticoids as an adjuvant to long-acting local anesthetics may decrease postoperative opioid use and reduce pain scores as compared to FPBs with LB or surgical site local anesthetic infiltration. Bupivacaine or ropivacaine combined with DXP and MPA is a safe and effective alternative to LB for FPBs in laparoscopic nephrectomy.

## Introduction

Laparoscopic nephrectomy has quickly been adopted as the standard of treatment for renal mass management [1]. These patients traditionally receive local anesthetic infiltration by surgeons or recently perioperative ultrasound-guided fascial plane blocks (FPBs) with the goal of improving pain control, decreasing opioid use, and decreasing hospital length of stay [2-5]. Nevertheless, studies have generated conflicting reports on whether FPBs are indicated in laparoscopic procedures and which cost-effective combinations of local anesthetics most improve the analgesic duration and recovery outcomes [6-8].

Several classic and emerging FPBs are used to improve pain after abdominal surgery, including transversus abdominus plane (TAP) blocks, rectus sheath (RS) blocks, and anterior quadratus lumborum (aQL) blocks [9, 10, 11]. In laparoscopic nephrectomy, TAP blocks may improve the quality of postoperative recovery and postoperative pain control [12] and may also reduce the incidence of developing chronic pain following laparoscopic nephrectomy [13]. Similarly, a recent meta-analysis supports using aQL blocks in laparoscopic nephrectomy to reduce opioid consumption [2].

Perineural glucocorticoids, including dexamethasone sodium phosphate (DXP) and methylprednisolone acetate (MPA), are used off-label in combination with long-acting local anesthetics, bupivacaine or ropivacaine, to prolong the duration of analgesia of neuraxial and peripheral nerve blocks alike [14-16]. Similarly, liposomal bupivacaine (LB), extended-release bupivacaine, was introduced in 2011 to increase the duration of interscalene brachial plexus blocks and TAP blocks [17, 18]. Recent data suggest brachial plexus blocks using bupivacaine with dexamethasone may provide similar pain control to bupivacaine with LB after shoulder surgery [19, 20]. To our knowledge, no studies have compared the efficacy of FPBs using bupivacaine with LB to FPBs using bupivacaine or ropivacaine with a glucocorticoid combination of DXP and MPA.

This retrospective study was designed to assess the postoperative analgesic efficacy of preoperative ultrasound-guided FPBs in patients undergoing laparoscopic non-donor nephrectomy. Patients received FPBs with either bupivacaine plus LB, bupivacaine or ropivacaine plus glucocorticoids (DXP and MPA), or surgical site local anesthetic infiltration by the surgeon without FPBs. The authors hypothesized that patients who received FPBs with bupivacaine or ropivacaine plus DXP and MPA would have the same analgesic outcomes as those who received bupivacaine plus LB, and both block groups would have better analgesic outcomes than those who received surgical site local anesthetic infiltration without FPBs. The primary outcome measured was postoperative opioid use. Secondary outcomes included postoperative pain scores, hospital length of stay, and postoperative complications.

## Materials and methods

Study design and data sources

This study was exempt from institutional review board approval as a retrospective cohort analysis. All elective laparoscopic non-donor nephrectomies between January 2018 and December 2022 at two hospitals that are part of a multi-hospital healthcare system were reviewed. Non-donor radical and partial nephrectomy patients were divided into three groups: 1) those who received preoperative FPBs with bupivacaine or ropivacaine plus DXP and MPA without intraoperative local anesthetic infiltration by the surgeon; 2) those who received preoperative FPBs with bupivacaine plus LB without intraoperative local anesthetic infiltration by the surgeon; and 3) those who received intraoperative local anesthetic infiltration by the surgeon without preoperative FPBs. All FPB patients received aQL blocks, but a negligible portion of patients received additional FPB blocks, including TAP and/or RS blocks, at the very start of the study before the team was certain about the effectiveness of aQL blocks in nephrectomy. All blocks were performed under standard American Society of Anesthesiologists (ASA) monitors after receiving intravenous midazolam and fentanyl as needed for sedation. Patients in the glucocorticoid group received 10 milligrams (mg) of DXP and 80 mg of MPA perineurally. Patients in the LB group received 266 mg of LB perineurally. Patients in the local anesthetic infiltration group were given bupivacaine with LB or bupivacaine with non-LB adjuvants at the discretion of the surgeon.

Patient inclusion and exclusion criteria

Patients were included if they received preoperative ultrasound-guided FPBs with either bupivacaine or ropivacaine plus 10 mg DXP and 80 mg MPA, FPBs with bupivacaine plus 266 mg LB, or surgical site local anesthetic infiltration without FPB. Patients were excluded if they underwent open nephrectomy or additional surgical procedures, received other local anesthetic combinations or adjuvants in addition to FPBs, remained intubated postoperatively, received epidurals, or received remifentanil intraoperatively. No exclusions were made based on patient comorbidities.

Measurements and data handling

The primary independent variable was the medication used in the FPB (MPA and DXP vs. LB vs. no FPB with surgical site local infiltration). Demographic and baseline covariates, including age, sex, body mass index (BMI), ASA status, hospital site, preoperative opioid use (NARX score), surgical time, and past medical history, including cardiac disease, pulmonary disease, psychiatric disease, diabetes mellitus, and obstructive sleep apnea, were documented.

The primary endpoint was postoperative opioid use at 0-48 hours, 0-12 hours, 12-24 hours, and 24-48 hours. Opioid use was totaled and converted to oral milligram morphine equivalents (MME) using an opioid conversion calculator [21]. Secondary endpoints were postoperative pain scores at 0-12 hours, 12-24 hours, and 24-48 hours; hospital length of stay; and postoperative complications (infection, bleeding, delirium, respiratory depression requiring naloxone, and unplanned intensive care unit admission) during the first 30 days.

Statistical analysis

Continuous variables are presented as mean (standard deviation) or median (interquartile range), as appropriate, while categorical variables are presented as N (percentage). Various statistical tests, such as analysis of variance (ANOVA), Kruskal-Wallis test, Chi-square test, or Fisher's exact test, were employed for group comparisons based on the type of data. The specific test used for each comparison is indicated in the table footnote.

Propensity scores (PS) were estimated for each patient using a vector-generalized linear model with a logit link. The model included all covariates listed in Table 1. The estimation was performed using the Vector Generalized Linear and Additive Models (VGAM) package v1.1-8 in R. Covariate balancing treatment weights (CBTW) were applied as numeric weights to subsequent outcome analyses. The Covariate Balancing Propensity Scor (CBPS) package v0.23 in R was used to implement covariate balancing. The balance of covariates was carefully assessed, and a standardized mean difference plot was used to evaluate the improvement in covariate balance after weighting.

Different CBTW regression models were employed based on the outcome variable of interest. Because the MME variable was skewed to the right, Poisson regression with a log link was used to model its distribution using its rounded value. Logistic regression with a logit link was used for the opioid-free outcome, and linear regression was used for pain scores (either at rest or during activity). Repeated measures of MME and pain scores obtained during the 0-12 hour, 12-24 hour, and 24-48 hour intervals were analyzed using the generalized estimating equations (GEE) method. The models included time, treatment group, and time by treatment group interaction term. An independent within-subject working correlation structure was assumed, but robust standard errors were used for statistical inference. The CBTW Cox model was used to analyze the length of hospital stay. Treatment effects were expressed in terms of adjusted mean ratio for MME, adjusted odds ratio for opioid-free, adjusted mean difference for pain score, and adjusted hazard ratio for hospital discharge (aHR>1 corresponds to faster hospital discharge).

All statistical analyses were performed using R v4.3.0 in RStudio v2023.03.1 (R Foundation, Vienna, Austria). A two-sided p-value of <0.05 was considered statistically significant.

## Results

Baseline demographics and clinical characteristics

**Figure 1:**
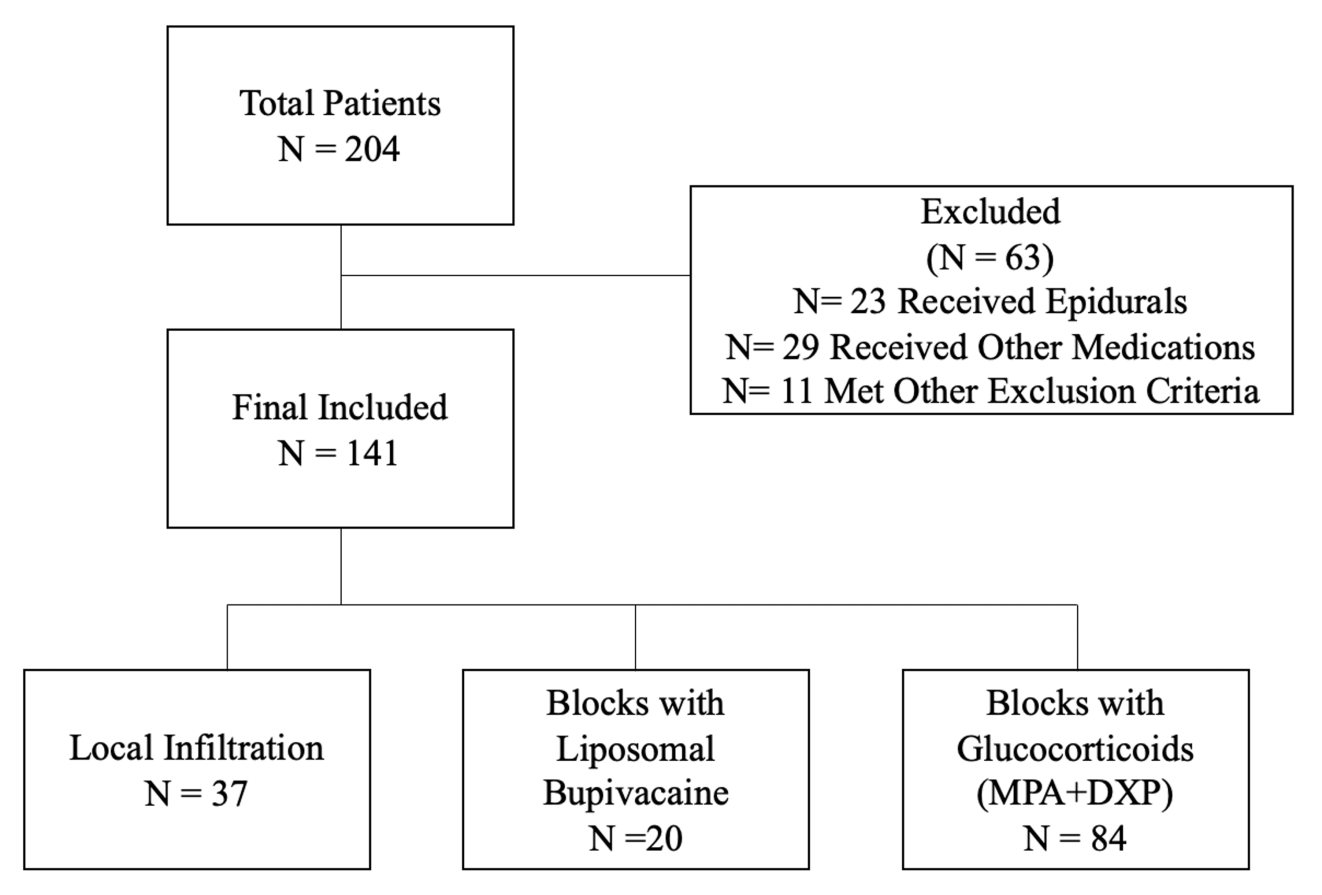
Flowchart of screened and excluded patients. MPA: methylprednisolone acetate; DXP: dexamethasone sodium phosphate

**Figure 2:**
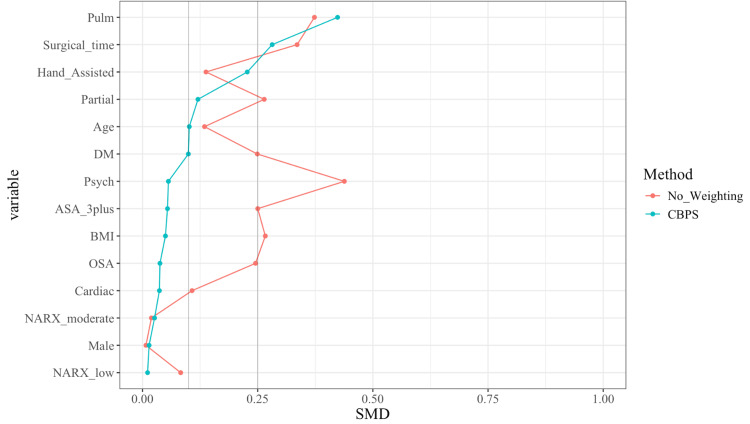
Standardized mean difference plot pulm: pulmonary; OSA: obstructive sleep apnea; psych: psychiatrical; NARX: narcotic (NARX) score; BMI: body mass index; ASA: American Society of Anesthesiologists; DM: diabetes mellitus; CBPS: Covariate Balancing Propensity Score

A total of 204 laparoscopic non-donor nephrectomy patients were screened; 141 patients were included in the analysis, and 63 patients were excluded based on exclusion criteria. Twenty patients were included in the LB group, 84 in the glucocorticoid group, and 37 in the local anesthetic infiltration group (Figure 1). Except for hospital site and a history of psychiatric disorders, no significant difference was found in demographic characteristics (age, sex, BMI), procedure type (radical or partial nephrectomy), surgical duration, preoperative opioid use, or comorbidities (ASA status, cardiac disease, pulmonary disease, diabetes mellitus, obstructive sleep apnea) between the three groups (Table 1). The use of the inverse CBPS as a numerical weight further improved the group balances for demographic and clinical characteristics, as seen in the standardized mean difference plot (Figure 2).

**Table 1:** Demographic, comorbidities, and intraoperative data Note: Comparisons were based on (a) ANOVA, (c) Chi-square test, (f) Fisher's exact test, and (k) Kruskal-Wallis test. BMI (p=0.122 (a)), pulmonary (p=0.096 (f)), diabetes (p=0.134 (f)), psychiatric (p<.001 (f)), hospital campus (p<.001 (c)) and length of stay (p=0.142 (k)). All others had p>0.2.

Baseline and perioperative characteristics	Local infiltration	Liposomal bupivacaine	Dexamethasone/methylprednisolone	Overall
	(N=37)	(N=20)	(N=84)	
Age (years), Mean (SD)	60.9 (14.4)	63.8 (14.9)	62.5 (13.3)	62.3 (13.7)
Sex, N (%)				
Female	15 (40.5%)	8 (40.0%)	34 (40.5%)	57 (40.4%)
Male	22 (59.5%)	12 (60.0%)	50 (59.5%)	84 (59.6%)
Body mass index (BMI), Mean (SD)	33.0 (8.52)	30.9 (5.56)	30.1 (6.73)	30.9 (7.16)
American Society of Anesthesiologist (ASA) score, N (%)				
1 and 2	10 (27.0%)	4 (20.0%)	31 (36.9%)	45 (31.9%)
3 and 4	27 (73.0%)	16 (80.0%)	53 (63.1%)	96 (68.1%)
Comorbidities, N (%)				
Cardiac	25 (67.6%)	12 (60.0%)	57 (67.9%)	94 (66.7%)
Pulmonary	2 (5.4%)	5 (25.0%)	14 (16.7%)	21 (14.9%)
Diabetes	8 (21.6%)	5 (25.0%)	9 (10.7%)	22 (15.6%)
Obstructive sleep apnea	6 (16.2%)	1 (5.0%)	7 (8.3%)	14 (9.9%)
Psychiatric	12 (32.4%)	5 (25.0%)	6 (7.1%)	23 (16.3%)
Narcotic score (NARX), N (%)				
Low	34 (91.9%)	19 (95.0%)	79 (94.0%)	132 (93.6%)
Moderate-high	3 (8.1%)	1 (5.0%)	5 (6.0%)	9 (6.4%)
Nephrectomy type, N (%)				
Partial	19 (51.4%)	8 (40.0%)	50 (59.5%)	77 (54.6%)
Radical	18 (48.6%)	12 (60.0%)	34 (40.5%)	64 (45.4%)
Hospital campus, N (%)				
Medical campus A	8 (21.6%)	19 (95.0%)	39 (46.4%)	66 (46.8%)
Medical campus B	29 (78.4%)	1 (5.0%)	45 (53.6%)	75 (53.2%)
Surgical technique, N (%)				
Hand-assisted	5 (13.5%)	4 (20.0%)	18 (21.4%)	27 (19.1%)
Length of surgery (hours), Median (IQR)	309 (254, 389)	286 (247, 320)	293 (230, 333)	296 (237, 347)

Primary and secondary outcomes

**Figure 3:**
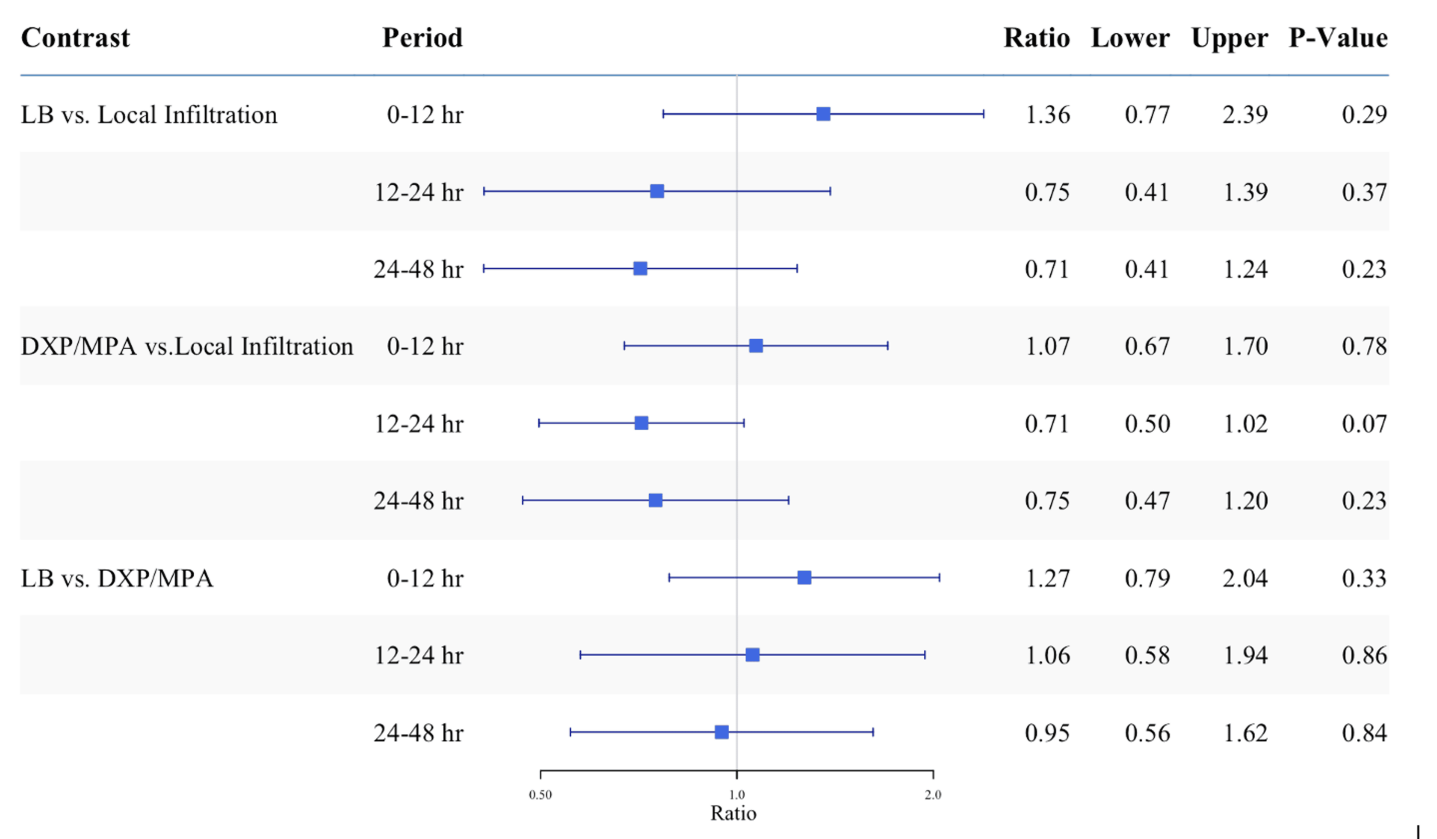
Postoperative opioid use in oral milligram morphine equivalents (MME) displayed as mean ratio P = 0.059 for the period by group interaction. Contrast estimates were obtained from outcome models with observations weighted by inverse covariates balancing propensity score. LB: liposomal bupivacaine; DXP/MPA: dexamethasone sodium phosphate and methylprednisolone acetate; hr: hour

The amount of perioperative MME (including preoperative opioids used for block placement and intraoperative opioids) was slightly higher in patients who received FPBs compared to patients who received surgical site local anesthetic infiltration without FPBs, as most block patients received fentanyl for sedation per institutional protocol (LB: CBTW-adjusted mean ratio 1.25 (1.00~1.56), p=0.048; DXP and MPA: 1.18 (1.02~1.37), p = 0.024; Table 2). There was no significant difference in perioperative MME between the LB and glucocorticoid groups (p=0.573; Table 2). Postoperatively, there was a trend toward decreased opioid requirements at 12-24 hours and 24-48 hours in patients who received ultrasound-guided FPBs with either LB or glucocorticoids when compared to patients who received local anesthetic infiltration (time and group interaction: p = 0.059; Figure 3). The glucocorticoid group had no difference in the cumulative 48-hour opioid dosage postoperatively, compared to the LB group (CBTW adjusted mean ratio: 0.91, (95% CI: 0.05~15.97); p = 0.948). Nevertheless, patients in the glucocorticoid group were more likely to be opioid-free 12-48 hours postoperatively compared to patients in the local anesthetic infiltration group (12-24 hours: CBTW-adjusted odds ratio 2.68 (0.91~7.91); p = 0.073; 24-48 hours: 3.87 (1.21~12.42); p = 0.023). Overall, patients in the glucocorticoid group received 30% fewer opioids 0-48 hours postoperatively compared to the local anesthetic infiltration group (30 MME vs. 44 MME, respectively; Table 2).

**Table 2:** Univariate comparisons for total perioperative opioid requirement in oral milligram morphine equivalents (MME) *p-value excludes missing values (f) Fisher's exact test and (k) Kruskal-Wallis test IQR: interquartile range

Opioid consumption	Local infiltration	Liposomal bupivacaine	Dexamethasone/methylprednisolone	p-value	Overall
	(N=37)	(N=20)	(N=84)		
Perioperative MME, median (IQR)	82 (58, 100)	98 (70, 117)	95 (74, 111)	0.186 (k)	90 (70, 110)
Postoperative MME, median (IQR)					
0-12 hours	10 (5, 20)	21 (6, 30)	11 (5, 25)	0.309 (k)	12 (5, 25)
12-24 hours	12 (5, 25)	10 (0, 20)	10 (0, 20)	0.356 (k)	10 (0, 20)
24-48 hours	15 (5, 33)	10 (0, 21)	6 (0, 25)	0.056 (k)	10 (0, 26)
Cumulative 0-48 hours	44 (15, 73)	41 (20, 70)	30 (10, 66)	0.382 (k)	35 (14, 70)
Opioid-free, N(%)					
0-12 hours	8 (21.6%)	5 (25.0%)	17 (20.2%)	0.869 (f)	30 (21.3%)
12-24 hours	7 (18.9%)	6 (30.0%)	26 (31.0%)	0.370 (f)	39 (27.7%)
24-48 hours	5 (13.9%)	6 (30.0%)	34 (40.5%)	0.017 (f)	45 (32.1%)
Cumulative 0-48 hours	2 (5.4%)	2 (10.0%)	12 (14.3%)	0.458 (f)	16 (11.3%)

**Figure 4:**
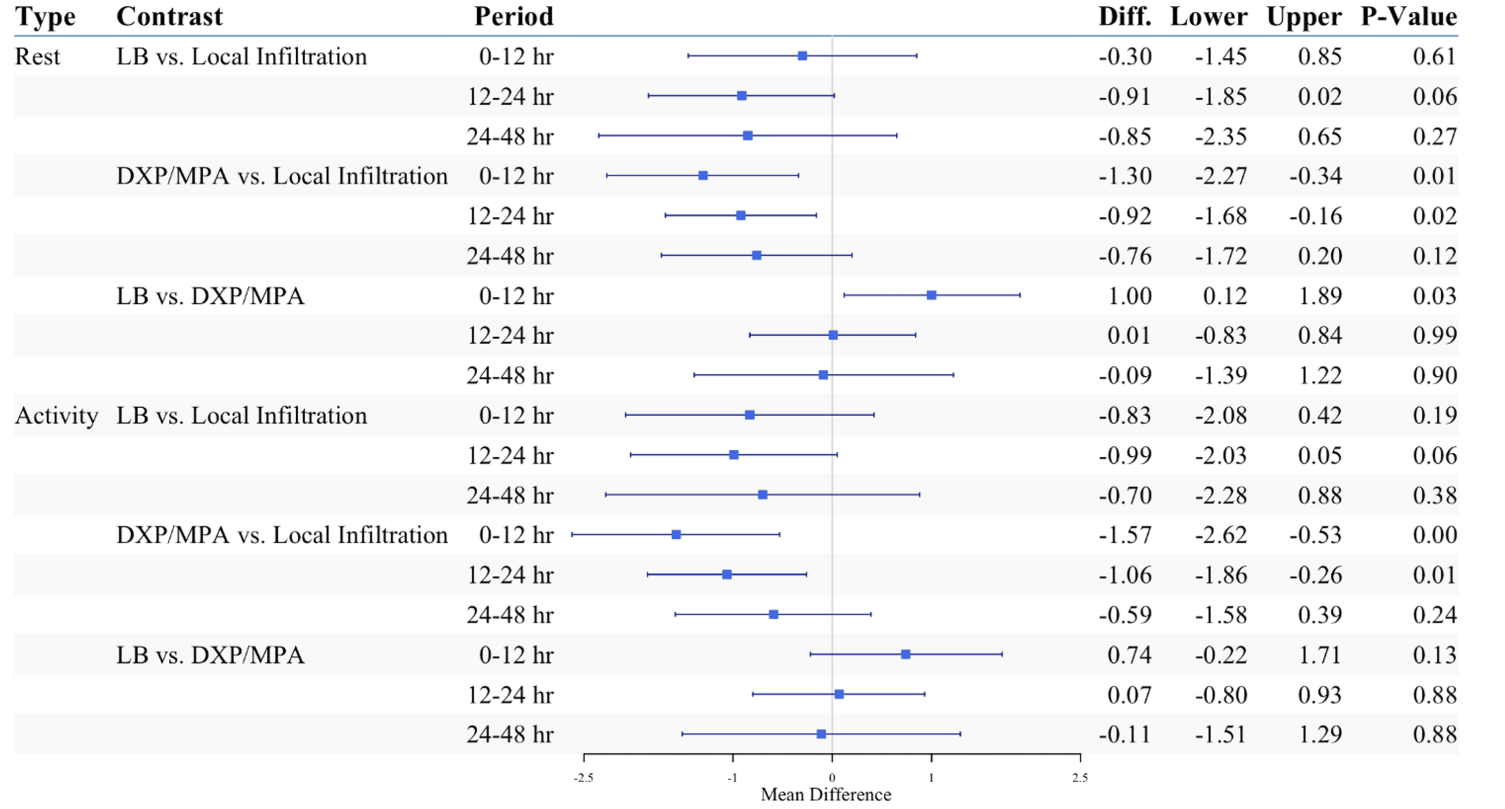
Postoperative pain scores at rest and with activity displayed as mean difference. P <0.01 for period and group in both types of pain scores Contrast estimates were obtained from outcome models with observations weighted by inverse covariates balancing propensity score. LB: liposomal bupivacaine; DXP/MPA: dexamethasone sodium phosphate and methylprednisolone acetate; hr: hour

Patients who received ultrasound-guided FPBs with glucocorticoids reported less pain at rest 0-12 hours and 12-24 hours postoperatively compared to those who received surgical site local anesthetic infiltration (p = 0.008 and p = 0.017, respectively; Figure 4). Likewise, patients in the LB group trended toward decreased pain scores at rest compared to patients in the local anesthetic infiltration group 12-24 and 24-48 hours, but this did not reach statistical significance (p = 0.06 and p = 0.27, respectively; Figure 4). Patients in the glucocorticoid group reported less pain at rest 0-12 hours postoperatively compared to patients in the LB group (p = 0.013, Figure 4). This difference was not observed at 12-24 or 24-48 hours postoperatively (p=0.990 and p=0.900, respectively; Figure 4). Overall, similar patterns were observed among the three groups for pain with activity (Figure 4).

There was no significant difference in postoperative complications among the groups in the first 30 days (p = 0.280, Table 3). Even though there is a trend toward decreased length of hospital stays in patients who received FPBs, especially for the glucocorticoid group, it failed to reach statistical significance (CBTW adjusted hazard ratio for earlier hospital discharge: 1.26 (0.08~20.94); p = 0.362). None of the patients experienced FPB-related complications, such as local anesthetic systemic toxicity or adverse drug effects.

**Table 3:** Univariate comparisons of postoperative pain scores at rest and with activity, adverse events, and length of stay * p-value excludes missing values (a) ANOVA, (f) Fisher's exact test, and (k) Kruskal-Wallis test IQR: interquartile range

Perioperative outcomes	Local infiltration (N=37)	Liposomal bupivacaine (N=20)	Dexamethasone/methylprednisolone (N=84)		p-value	Overall
Postoperative pain scores at rest, mean(SD)						
0-12 hours	4.50 (2.16)	4.33 (1.95)		3.28 (1.92)	0.006 (a)	3.72 (2.04)
12-24 hours	4.10 (2.07)	3.15 (2.10)		3.21 (1.88)	0.088 (a)	3.40 (1.98)
24-48 hours	3.98 (2.35)	3.11 (2.17)		2.96 (1.74)	0.086 (a)	3.23 (2.00)
Post-operative pain scores with activity, Mean(SD)						
0-12 hours	5.12 (2.36)	4.18 (2.41)		3.63 (2.15)	0.014 (a)	4.02 (2.29)
12-24 hours	4.52 (2.10)	3.38 (2.22)		3.50 (1.94)	0.054 (a)	3.70 (2.05)
24-48 hours	4.12 (2.30)	3.31 (2.22)		3.26 (1.95)	0.206 (a)	3.47 (2.09)
Length of stay (hours), median (IQR)	66 (46, 90)	59 (49, 73)		48 (28, 72)	0.055 (k)	51 (42, 74)
Adverse events, N (%)	2 (5.4%)	3 (15%)		5 (6%)	0.280 (f)	10 (7.1%)

## Discussion

This single-center propensity score-weighted retrospective study analyzed three groups of laparoscopic non-donor nephrectomy patients, including patients who received perioperative FPBs with bupivacaine or ropivacaine plus DXP and MPA, patients who received FPBs with bupivacaine plus LB, and patients who received surgical site local anesthetic infiltration without a FPB. Patients who received FPBs with glucocorticoids were statistically more likely to be opioid-free 12-48 hours postoperatively. They also reported statistically lower pain scores 0-12 hours postoperatively as compared to patients who received FPBs with bupivacaine plus LB or surgical site local anesthetic infiltration. Although there was a trend of approximately 25% decrease in total postoperative opioid use in the glucocorticoid group as compared to the LB group and a nearly 20% decrease in length of hospital stay, neither reached statistical significance. To the best of our knowledge, this is one of the first studies comparing outcomes in patients who received FPBs with glucocorticoids versus LB.

Cumulatively, patients in the glucocorticoid group received about 10 MME less than those in the LB group. This was not found to be statistically significant, nor is it likely to be clinically relevant. However, 30% of patients in the LB group and 40% in the glucocorticoid group were opioid-free 24-48 hours postoperatively, compared to 14% in the local infiltration group. This suggests that FPBs using LB or glucocorticoids both provide higher quality prolonged analgesia in laparoscopic non-donor nephrectomy patients compared to surgical site local anesthetic infiltration. Most FPB patients in this study were discharged 48-60 hours postoperatively, requiring little or no opioid prescriptions (data not shown).

At each time point, patients who received FPBs with LB or glucocorticoids trended toward lower pain scores at rest and with activity compared to patients who did not receive FPBs. Patients who received glucocorticoids had the lowest pain scores at rest and with activity 0-12 hours postoperatively. A significant difference in pain scores between the glucocorticoid and LB groups was only observed for the first 12 hours postoperatively, not at 12-24 or 24-48 hours postoperatively. Overall, this suggests that FPBs with LB or glucocorticoids provide excellent analgesia compared to surgical site local anesthetic infiltration. However, neither LB nor glucocorticoids appear to be superior to each other.

Liposomal bupivacaine has been shown to prolong the duration of FPBs in laparoscopic nephrectomy patients as well as other abdominal surgeries when compared to plain bupivacaine or placebo [15, 22]. Similarly, DXP has been shown to prolong the duration of FPBs in abdominal surgeries when added to bupivacaine or ropivacaine [23, 24]. This study confirms previous studies that show ultrasound-guided FPBs with DXP or LB provide superior analgesia compared to patients who do not receive FPBs. To the best of our knowledge, no studies have directly compared the analgesic efficacy of FPBs with LB versus glucocorticoids DXP and MPA. Nevertheless, studies comparing LB to glucocorticoids for brachial plexus blocks have shown conflicting results. Some suggest that brachial plexus blocks with bupivacaine plus DXP provide similar analgesia compared to LB after shoulder surgery [17]. Others demonstrate a significant decrease in “worst pain scores” and 72-hour opioid use in patients who received bupivacaine plus LB versus bupivacaine or ropivacaine plus DXP [25].

Intravenous (IV) DXP has been shown to decrease pain scores and postoperative nausea and vomiting (PONV) in donor nephrectomy patients without increasing surgical complications [26]. There are conflicting studies on the efficacy of IV versus perineurial glucocorticoids for prolonging the duration of extremity blocks [27]. Most recently, a new volunteer study demonstrated that perineural DXP prolongs the duration of nerve blocks more than IV DXP [28].

For the past several years, the study institute has used a combination of hydrophilic glucocorticoid (DXP), commonly used in acute pain management, and lipophilic glucocorticoid (MPA), commonly used in chronic pain management, to transition from continuous catheters to single-injection nerve blocks in ambulatory total knee replacements [29]. The goal of this study was to directly compare FPBs with glucocorticoids DXP and MPA to FPBs with LB in laparoscopic nephrectomy.

In summary, this is the first study demonstrating that the addition of glucocorticoids DXP and MPA to FPBs may serve as a cost-effective alternative to LB in laparoscopic nephrectomy. Overall, FPB patients who received DXP and MPA or LB had more effective pain control than those who received surgical site local anesthetic infiltration without FPBs. Patients who received FPBs with glucocorticoids may be more likely to remain opioid-free after 12 hours and have lower pain scores in the first 12 hours. This may result in improved patient satisfaction and decrease overall healthcare costs. A future multicenter prospective randomized control trial with a longer follow-up time beyond the perioperative period may be beneficial to evaluate the role of FPBs with DXP and MPA in combination with other surgeries.

Limitations

There are several limitations to this study. First, retrospective studies are not appropriate to test analgesic interventions in general because there is a lack of standard analgesic medications in that two long-acting local anesthetics, bupivacaine or ropivacaine, were used. Retrospective studies have inadequacies in data collection and the potential for confounding variables when evaluating opioid use and pain scores. Pain scores are inherently subjective, and retrospective studies rely on physician and nursing records. Fortunately, our electronic medical record tracks these pain scores, and our regional anesthesiology team evaluates nerve block patients on postoperative day one. Second, there was a significant imbalance between the size of the three groups and the hospital site of the surgeries, which is common in retrospective studies. We understand balancing study groups is crucial; therefore, study subjects were matched based on potential confounding factors in demographic and perioperative characteristics using propensity scores. Different covariate balancing treatment weight regression models were subsequently employed based on the outcome variable of interest. In the end, the groups had similar demographic data and propensity score weighting in statistical analysis. Third, this study was performed at a single large academic health system with a dedicated group of regional anesthesiologists, and all nephrectomies were performed by a small group of surgeons across both hospitals. This may limit the potential for confounding factors but may also limit its generalizability across institutions. Ideally, a future prospective study across multiple institutions could help eliminate group imbalance and control for confounding. Lastly, about 25% of patients in each group received four to 10 milligrams of IV DXP for PONV prophylaxis. Although equally distributed among the groups, this may confound the results. Any future studies should consider controlling for IV glucocorticoids.

## Conclusions

Fascial plane blocks provide more effective pain control than surgical local infiltration. Patients who received FSBs with a glucocorticoid combination, including DXP and MPA, may be more likely to have lower pain scores in the first 12 hours and remain opioid-free after 12 hours than LB in laparoscopic non-donor nephrectomy.
